# Lupeol inhibits osteosarcoma progression by up-regulation of HMGA2 via regulating miR-212-3p

**DOI:** 10.1186/s13018-020-01879-0

**Published:** 2020-09-03

**Authors:** Jinghua Zhong, Chunlei He, Fangtian Xu, Xianyun Xu, Lulin Liu, Mingjun Xu, Zheng Guo, Yili Wang, Jiahua Liao, Yonghong Li

**Affiliations:** 1 Department of Oncology, The First Affiliated Hospital of Gannan Medical University, Ganzhou, Jiangxi China; 2 Department of Orthopedics, The First Affiliated Hospital of Gannan Medical University, Ganzhou, Jiangxi China; 3 Basic Medical School, Gannan Medical University, Ganzhou, Jiangxi China; 4 Department of Oncology, The First Hospital of Tianmen City of Hubei Province, No. 1, East Renmin Avenue, Tianmen, 431700 Hubei China

**Keywords:** OS, Lupeol, miR-212-3p, HMGA2, Viability, Invasion, Apoptosis

## Abstract

**Background:**

Osteosarcoma (OS) is a common severe illness globally. Lupeol has been reported to participate in the pathophysiologic properties of various cancers, including OS. This study aimed to explore the effects of lupeol on proliferation, invasion, and apoptosis on OS cells and the underlying mechanism.

**Methods:**

The cell viability of OS cells was determined by 3-(4, 5-dimethyl-2-thiazolyl)-2,5-diphenyl-2-H-tetrazolium bromide (MTT) assay. The expression levels of miR-212-3p and high-mobility group AT-hook 2 (HMGA2) were detected by quantitative real-time polymerase chain reaction (qRT-PCR) in OS cells. The cell apoptosis and invasion were detected by flow cytometry and transwell invasion assays, respectively. The functional target of miR-212-3p was predicted by online software and confirmed by luciferase reporter assay. The protein level of HMGA2 was measured by western blot analysis.

**Results:**

Lupeol suppressed cell viability and invasion, and promoted apoptosis by upregulating the expression of miR-212-3p in OS cells. Knockdown of miR-212-3p restored the anti-tumor effect of lupeol. Interestingly, miR-212-3p directly targeted HMGA2 and suppressed its expression. Moreover, HMGA2 reversed the inhibited impact on viability and invasion, and the promoted effect on apoptosis induced by upregulation of miR-212-3p. Also, lupeol administration exerts its anti-tumor effect by overexpression of miR-212-3p to suppress the expression of HMGA2 in OS cells.

**Conclusion:**

Lupeol inhibited OS progression by modulating the miR-212-3p/HMGA2 axis in vitro.

## Introduction

Osteosarcoma (OS) is the most frequent mesenchymal sarcoma derived from the bone matrix synthesized and secreted by neoplastic cells [1]. Although the survival rate of patients with OS significantly improved by modern treatment [2, 3], 25–35% of patients with initial non-metastatic disease subsequently metastasized, which remains the leading cause of death [4]. Over the two decades, there are many accumulating evidence manifest that unbalances of molecule-regulated participated in the pathological progress of OS. Whereas, the latent mechanism of OS still needs to explore. Thus, it is essential to find novel biomarkers for optimizing therapeutic strategies and predictions for clinical outcomes.

Lupeol (Lup-20(29)-en-3β-ol), a significant lupane-type triterpene existed in natural plants of garden stuff [5, 6], with pharmacological effects of anti-inflammatory [7], and anticancer activities [8, 9]. It has raised much attention due to its hypo-toxicity and a wide range of therapeutic effects. In recent years, the impact of lupeol on chemoprevention and potential preventing from various cancers has been widely demonstrated, such as prostate cancer [10], breast cancer [9], squamous cell carcinoma [11], melanoma [12], and OS [13]. A previous paper showed that lupeol inhibited migration and invasion of OS cells by regulating p38/MAPK and PI3K/Akt signaling pathways in vitro [13]. Whereas, the precise mechanism of lupeol involved in the progression of OS remains plausible.

MicroRNAs (miRNAs) are a kind of endogenous short RNAs containing about 22 nucleotides in length. Evidence is increasingly supporting that some miRNAs exert tumor-suppressed roles by targeting and inhibiting multiple oncogenes’ expression. Deregulated miRNAs could be observed in diverse types of cancers and were crucial in promoting or suppressing the progression of malignant tumors [14]. Therefore, to explore novel miRNAs and defined their function are necessary for modern cancer therapies. A previous study indicated that the marked low-expression of miR-212-3p was observed in OS tissues. Additionally, it has been proved that miR-212-3p restrains OS cell proliferation and invasion via the sex-determining region Y-box 4 (Sox4) [15]. However, the role of miR-212-3p in OS and the underlying regulatory mechanism have not been entirely illuminated.

High-mobility group AT-hook 2 (HMGA2) is a transcription factor that belongs to the non-histone chromosomal high-mobility group (HMG) protein family and is typically correlated with gene expression by remodeling the chromatin state [16]. Changes of HMGA2 have been investigated in human mesenchymal tumors, pleomorphic adenomas, and OS [17]. A previous study indicated that miR-490-3p expression was negatively correlated with HMGA2 in OS, and the tumor-suppressed function of miR-490-3p could be reversed by overexpression of HMGA2 in OS cells [18].

This study demonstrated the anti-cancer function of lupeol in the viability, apoptosis, and invasion of OS cells, and its regulatory mechanism in OS progression was also explored.

## Materials and methods

### Cell culture and chemicals treatment

MNNG/HOS and MG-63cells were bought from Cell Bank of the Chinese Academy of Sciences (Shanghai, China), MNNG/HOS and MG-63 cells were cultured as described previously [19], and cells were plated in 24-well plate for 24 h before transfection.

Moreover, Lupeol (Sigma-Aldrich, St. Louis, MO, USA) was re-suspended with ethyl alcohol to the concentration of 30 mmol/L mother liquor preparation. It would be diluted in dimethyl sulfoxide (DMSO; Solarbio, Beijing, China) at a 1:1 ratio. The final concentrations were diluted by cell culture medium for preparation.

### Cell transfection

MiR-212-3p mimics or inhibitor, HMGA2 expression plasmid (pcDNA-HMGA2), and their negative controls were obtained from Ribobio Co. (Guangzhou, China). The oligos or plasmids were transfected into the cells with following the instruction of Lipofectamine 3000 (Thermo Fisher Scientific, Waltham, MA, USA).

### 3-(4, 5-dimethyl-2-thiazolyl)-2, 5-diphenyl-2-H-tetrazolium bromide (MTT) assay

MTT kit (Sigma, St Louis, MO, USA) was applied to assess the cell viability. After 48 h of transfection, untransfected or transfected MNNG/HOS and MG-63 (3 × 10^4^ cells/well) cells were treated with various concentrations of lupeol (0, 10, 20, 30 μM) for 12 h, 24 h, or 48 h. At the indicated time points after treatment, each well was added with 20 μL of MTT reagent, and cells were incubated for another 4 h. Subsequently, cells were collected and intracellular formazan crystals were dissolved with 200 μL of DMSO (Solarbio, Beijing, China) [20]. Finally, the absorbance at 570 nm was detected with a microplate reader (Thermo Labsystems, Waltham, MA, USA).

### RNA isolation and quantitative real-time polymerase chain reaction (qRT-PCR) assay

Total RNA in cells was isolated using TRIzol reagent (Thermo Fisher Scientific), according to the previous description [21]. Then, All-in-One™ miRNA Prime Script™RT reagent kit (Takara, Shiga, Osaka, Japan) was used for reverse-transcription according to manufacturer’s instructions. qPCR was performed in a 20 μL total reaction volume comprised of 10 μL of SYBR Green qPCR Master Mix (2×) (Bio-Rad Laboratories, Lnc., Hercules, CA, USA), 1 μL of each gene-specific primer, 2 μL of cDNA templates, and 6 μL of PCR-grade water. Reactions were conducted on the 7500 Fast Real-Time PCR system (Thermo Fisher Scientific) in line with manufacturer’s protocol: denaturation at 94 °C for 2 min, 94 °C for 30 s, 54 °C for 30 s, 72 °C for 35 s, 30 cycles. The relative expressions were calculated by the 2^−ΔΔCt^ method and normalized to internal U6 small nuclear RNA (U6-snRNA, for miRNA) and GAPDH (for mRNA). The primers for miR-212-3p and U6 were purchased from Sangon Biotech (Shanghai, China). Primer sequences (5’-3’) of HMGA2 and GAPDH for qPCR were listed as follows: HMGA2-F (ATGAGCGCACGCGGTGAGGGC), HMGA2-R (GTTAGAAGACTCAAAGGAACAG), GAPDH-F (AAGCTGGTCATCAATGGGAAAC), GAPDH-R (ACCCCATTTGATGTTAGCGG).

### Flow cytometry assay

After washed with iced-phosphate buffered saline (PBS), cells were resuspended in binding buffer. AnnexinV-fluorescein isothiocyanate propidium iodide (AnnexinV-FITC/PI) kit (BD Pharmingen, San Diego, CA, USA) was used to strain the cells according to the instructions. The samples were verified by flow cytometry (BD Biosciences, San Jose, CA, USA).

### Transwell invasion assay

Transwell assay was employed to evaluate cell invasion. The upper chamber coated with Matrigel (Corning Life Sciences, Corning, NY, USA) was added 100 μL of serum-free medium containing cells (1 × 10^5^ cells per well). Cell medium with 10% serum was added into the basolateral chamber. After 24 h of incubation at 37 °C, cells located on the lower surface of the upper chamber was attached with paraformaldehyde (PFA; Sigma) and stained with crystal violet. Cells were analyzed under a microscope.

### Luciferase assay

After cells were cultured for 24 h, the wide-type and mutated HMGA2 3′UTR pMIR-REPOR luciferase vector (OBio Biology, Shanghai, China) were constructed and co-transfected with miR-212-3p mimic or negative control by using Lipofectamine 3000 (Thermo Fisher Scientific). The Dual-Luciferase Reporter AssaySystem (Promega, Madison, WI, USA) was used to detect the luciferase activities at 48 h post-transfection. Renilla luciferase served as an internal control.

### Western blot analysis

Total proteins were isolated by RIPA buffer (Solarbio, Beijing, China) and quantified using a NanoDrop 3000 (Thermo Fisher Scientific). After subjected to Sodium dodecyl sulfate polyacrylamide gel electrophoresis (SDS-PAGE), protein samples were transferred onto polyvinylidene fluoride membranes (Millipore, Bradford, MA, USA). Next, 5% fat-free milk buffer was used to block the membranes for 2 h at 37 °C. And then primary antibodies rabbit-anti-HMGA2 (1:500; Cell Signaling Technology, Danvers, MA, USA) or rabbit-anti-human GAPDH (1:1000; Cell Signaling Technology) was used to incubate the membranes overnight at 4 °C. The membranes were then washed with TBST and incubated with secondary antibody marked goat anti-rabbit IgG horseradish peroxidase (HRP) (1:2000; Cell Signaling Technology). After washing with TBST, the blots were detected using an ECL detection kit (Pierce Biotech, Rockford, IL, USA) and analyzed by Image Pro-Plus 6.0 software.

### Statistical analysis

All data were analyzed by the SPSS 21.0 software and expressed as mean ± standard deviation (SD) with at least three repeats independently. One-way analysis of variance (ANOVA) with Tukey’s tests was used to compare the difference among multiple groups. The data meet the requirements of the normal distribution. A *P* value < 0.05 was regarded as statistically significant.

## Results

### Lupeol repressed cell viability and upregulated the expression of miR-212-3p in OS cells

**Fig. 1:**
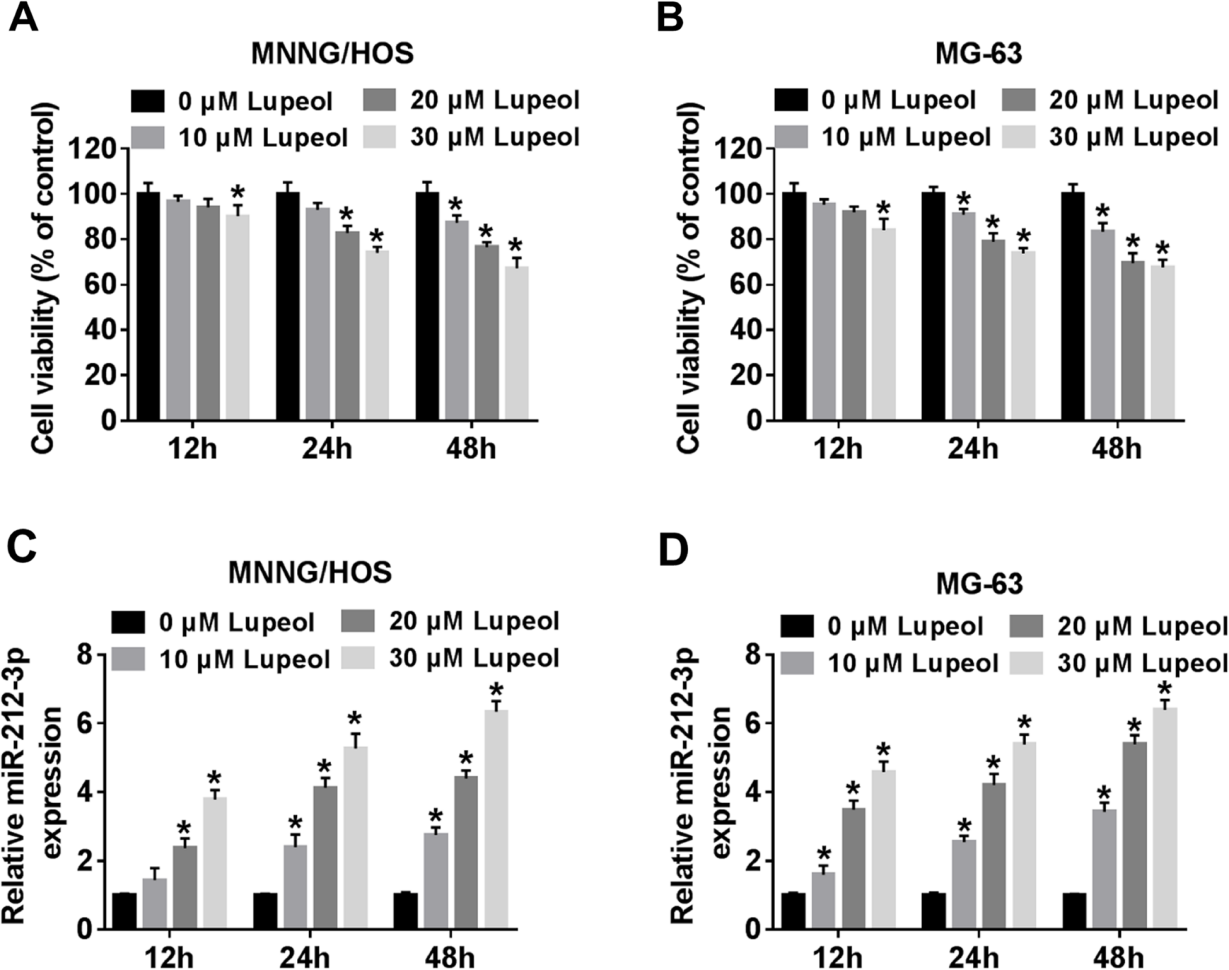
Lupeol modulates cell viability and is related to miR-212-3p. MNNG/HOS and MG-63 cells (1 × 10^5^cells/well) were incubated with lupeol (0μM, 10 μM, 20 μM, and 30 μM) for 12 h, 24 h, and 48 h. Cells were harvested for cell viability detection as depicted in Materials and methods. **a**, **b** MTT assay was used to detected cell viability of MNNG/HOS and MG-63 cells. **c**, **d** The expression levels ofmiR-212-3p in MNNG/HOS and MG-63 cells were tested by qRT-PCR. ^*^P < 0.05, *n* = 3

To detect the effects of lupeol on cytotoxicity, MNNG/HOS and MG-63 cells were incubated with lupeol at various concentrations (0 μM, 10 μM, 20 μM, and 30 μM) for 12 h, 24 h, and 48 h. We found that cell viability was negatively related to lupeol concentration (Fig. 1a, b). Based on these findings, we selected 20 μM lupeol (24 h treatment) [13] for further studies. QRT-PCR was performed to explore the expression of miR-212-3p in MNNG/HOS and MG-63 cells treated with lupeol. The analysis showed that the expression level of miR-212-3p was significantly increased as the concentration elevated (Fig. 1c, d). From these data, it could be deduced that lupeol repressed viability and upregulated the expression of miR-212-3p in OS cells in vitro.

### Lupeol, as well as overexpression of miR-212-3p suppressed cell viability, invasion, and promoted apoptosis in OS cells

**Fig. 2:**
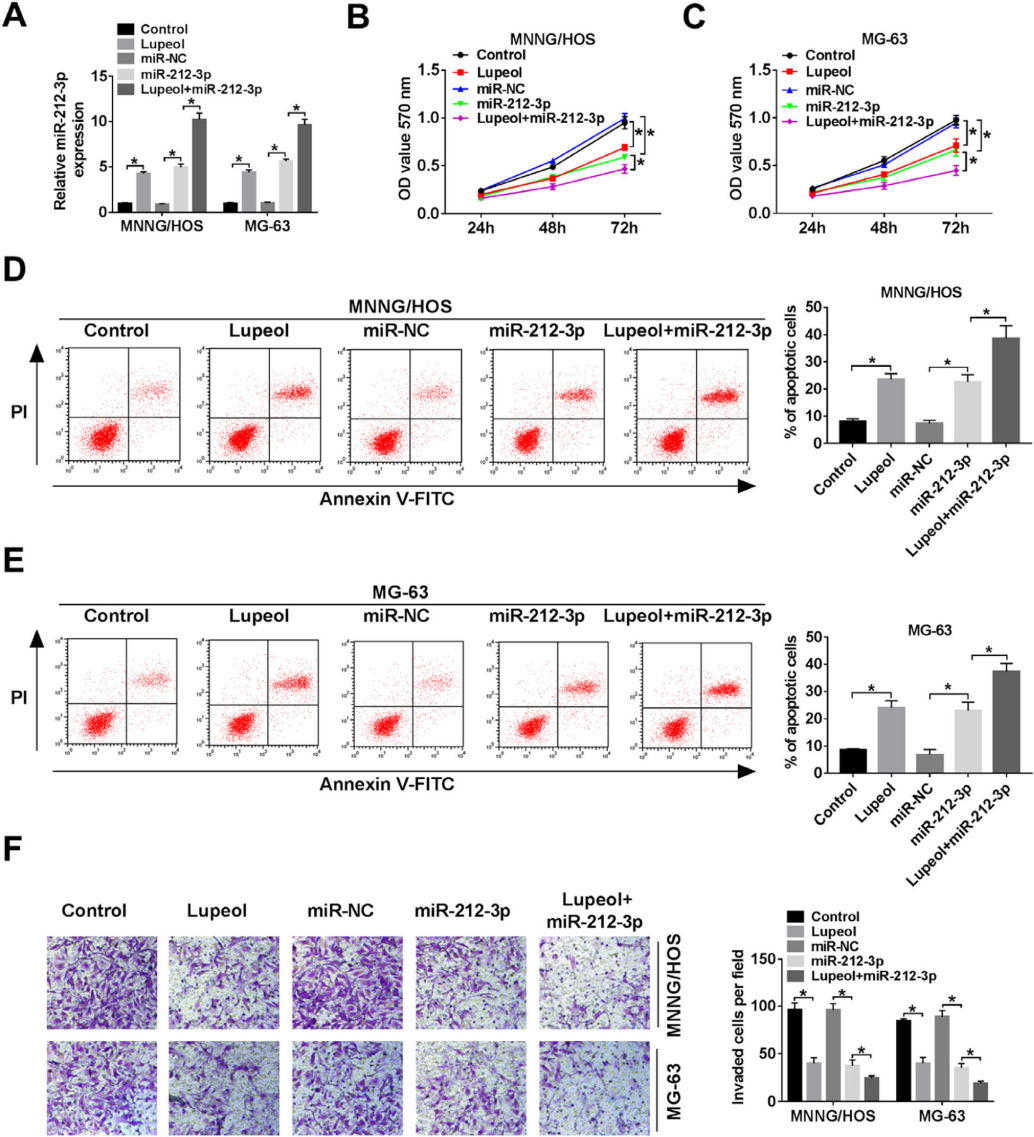
Lupeol, as well as overexpression of miR-212-3p, inhibited cell proliferation and invasion promoted apoptosis in osteosarcoma cells. **a** The miR-212-3p expression in MNNG/HOS and MG-63 cells was tested by qRT-PCR. **b**, **c** MTT assay was used to detect cell viability of MNNG/HOS and MG-63 cells with overexpression of miR-212-3p at the indicated time points. **d**, **e** Flow cytometry was used to detect apoptosis of MNNG/HOS and MG-63 cells after transfection for 24 h. **f** Transwell invasion assay was performed to determine the numbers of invaded cells. ^*^P < 0.05, *n* = 3

To investigate the functional effects of lupeol and miR-212-3p in OS, MNNG/HOS and MG-63 cells were transfected with miR-NC ormiR-212-3p. The result showed that lupeol management and the transfection of miR-212-3p caused a high expression level of miR-212-3p in MNNG/HOS and MG-63 cells. Interestingly, both overexpression of miR-212-3p and lupeol treatment enhanced mRNA expression of miR-212-3p in cells (Fig. 2a). MTT assay indicated that the viability of OS cells was significantly inhibited by lupeol treatment or overexpression of miR-212-3p (Fig. 2b, c). Flow cytometry assay demonstrated that lupeol treatment and overexpression of miR-212-3p led to an apparent increase in apoptosis rates in two OS cells (Fig. 2d, e). In addition, transwell assay revealed that lupeol treatment and overexpression of miR-212-3p could inhibit cell invasion of OS cells (Fig. 2f). These data above suggested that both lupeol and overexpression of miR-212-3p exerted an effect on inhibiting viability and invasion, promoting apoptosis on OS in vitro.

### Lupeol regulated viability, apoptosis, and invasion by up-regulating miR-212-3p

**Fig. 3:**
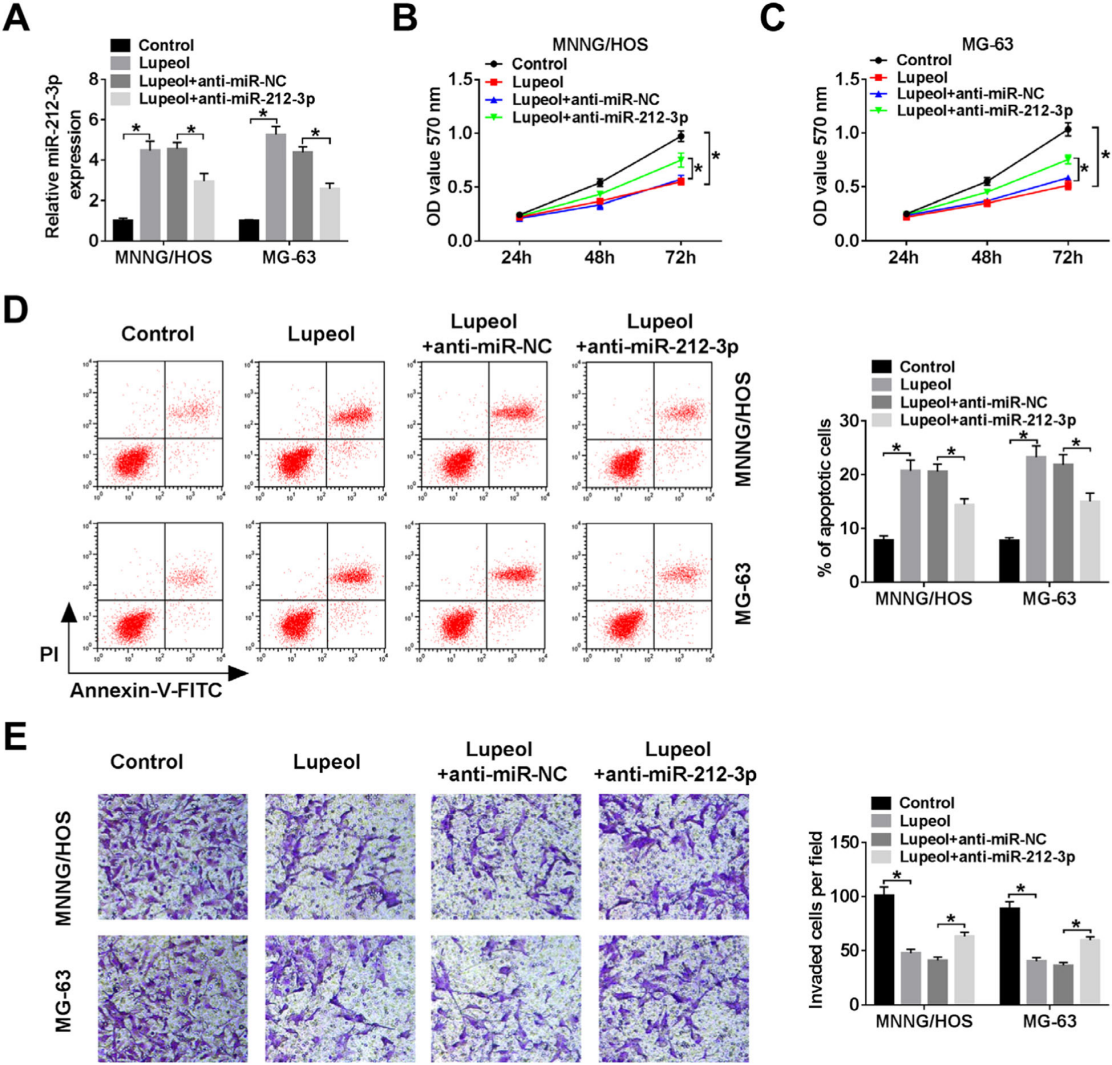
Lupeol regulated proliferation, apoptosis, and invasion by targeting miR-212-3p. **a M**iR-212-3p level was tested by qRT-PCR in MNNG/HOS and MG-63 cells. **b**, **c** MTT assay was used to demonstrate cell viability in MNNG/HOS and MG-63 cells silenced miR-212-3p in the different time points. **d** Flow cytometry was used to detect apoptosis of MNNG/HOS and MG-63 cells after transfection for24 h. **e** Transwell invasion assay was used to analyze the numbers of invaded cells. ^*^P < 0.05, *n* = 3

To verify the mechanism by which lupeol regulating apoptosis and invasion and figure out the relationship between lupeol and miR-212-3p in OS cells, MNNG/HOS and MG-63 cells were transfected with anti-miR-NC or anti-miR-212-3p, respectively. As shown in Fig. 3a, the expression level of miR-212-3p was downregulated in the Lupeol + anti-miR-212-3p group, compared with the negative control group. MTT and transwell assay demonstrated that viability and invasion were significantly inhibited by lupeol, and its effect could be overturned by knockdown of miR-212-3p (Fig. 3b, c, e). Flow cytometry analysis implied that miR-212-3p silencing reduced the high apoptosis rate resulting from lupeol administration (Fig. 3d). These results above uncovered that lupeol could regulate cell viability, apoptosis, and invasion by upregulatingmiR-212-3p in vitro.

### MiR-212-3p directly targeted HMGA2 and suppressed its expression

**Fig. 4:**
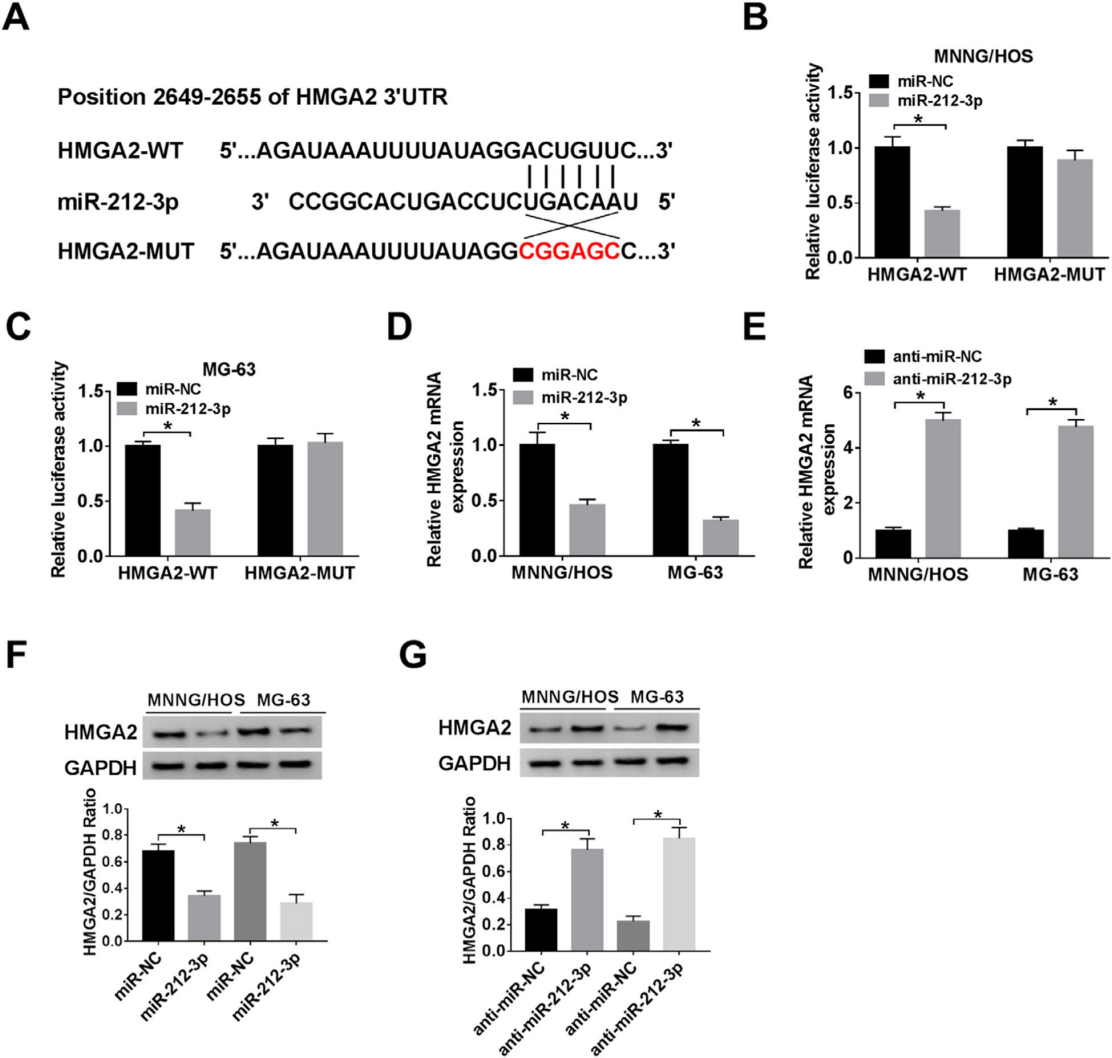
Identification of HMGA2 targeting miR-212-3p. **a** The binding sites between miR-212-3p in HMGA2 were predicted by starBase v2.0. **b**, **c** The relative luciferase activity of MNNG/HOS and MG-63 cells co-transfected with the miR-212-3p and pMIR-REPOR-SNHG1-WTor pMIR-REPOR-SNHG1-mut was measured. **d**, **f** The mRNA and protein expression levels of miR-145-5p on MNNG/HOS and MG-63 cells transfected with miR-NC andmiR-212-3p overexpression vector. **e**, **g** The mRNA and protein expression of HMGA2 was evaluated after transfection the miR-145-5p inhibitor into MNNG/HOS and MG-63 cells. ^*^P < 0.05, *n* = 3

By the TargetScan, bioinformatics software online, we identified that HMGA2 was a potential target of miR-212-3p. The binding site between HMGA2 and miR-212-3p locate at its 3′-UTR of HMGA2 with binding site 5′-ACUGUUC-3′ (Fig. 4a). To explore the potential interaction, the pMIR-REPOR-HMGA2-WT and the pMIR-REPOR-HMGA2-MUT plasmids were constructed. The luciferase reporter assay demonstrated that luciferase activity was obviously reduced in cells co-transfected with miR-212-3p and pMIR-REPOR-HMGA2-WT, while had no evident change in cells transfected with miR-212-3p and pMIR-REPOR-HMGA2-WT (Fig. 4b, c). We also detected the mRNA and protein levels of HMGA2 in OS cells with overexpression or knockdown of miR-212-3p. The results showed that HMGA2 was significantly decreased when cells were transfected with miR-212-3p, whether at the mRNA level (Fig. 4d) or protein level (Fig. 4f). By contrast, HMGA2 expression was significantly raised when cells were transfected with anti-miR-212-3p at both mRNA levels (Fig. 4e) and protein level (Fig. 4g).

### MiR-212-3p regulated viability, apoptosis, and invasion of OS cells by regulating HMGA2

**Fig. 5:**
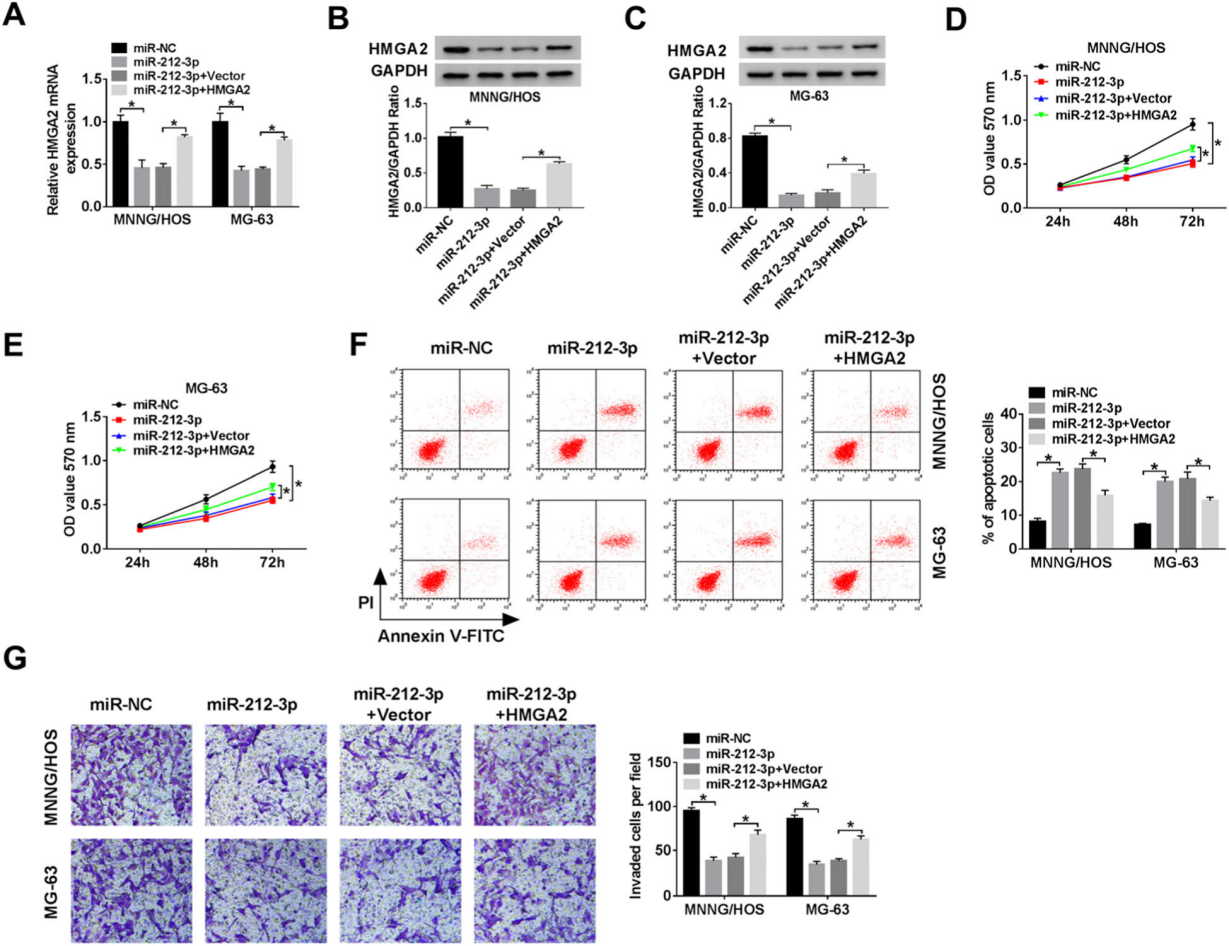
MiR-212-3p suppressed proliferation, apoptosis, and invasion in osteosarcoma cells by regulating HMGA2 in vitro. MNNG/HOS and MG-63 cells were transfected with miR-NC, miR-212-3p, miR-212-3p + Vector or miR-212-3p + HMGA2. **a**–**c** QRT-PCR and western blot were used to evaluate mRNA and protein expression level of HMGA2 separately. **d**, **e** Cell viability was detected by the MTT assay. **f**, **g** Flow cytometry and transwell invasion assays were used to detect apoptosis rate and the numbers of invaded cells differentially. ^*^P < 0.05, *n* = 3

To investigate the functional roles of HMGA2 in tumor cells, HMGA2 over-expressing vector was constructed and co-transfected into MNNG/HOS and MG-63 cells along with miR-212-3p. Cells co-transfected with miR-212-3p and HMGA2 exhibited high expression of HMGA2 at mRNA level (Fig. 5a) and protein level (Fig. 5b, c). In addition, we found that overexpression of HMGA2 restored the down-regulated effect on viability (Fig. 5d, e) and invasion (Fig. 5g) and upregulated effect on apoptosis induced by miR-212-3p (Fig. 5f). Taken together, the miR-212-3p exerts a cancer suppressor role in OS partially by inhibiting its target gene HMGA2.

### Lupeol inhibited the expression of HMGA2 by upregulating miR-212-3p

**Fig. 6:**
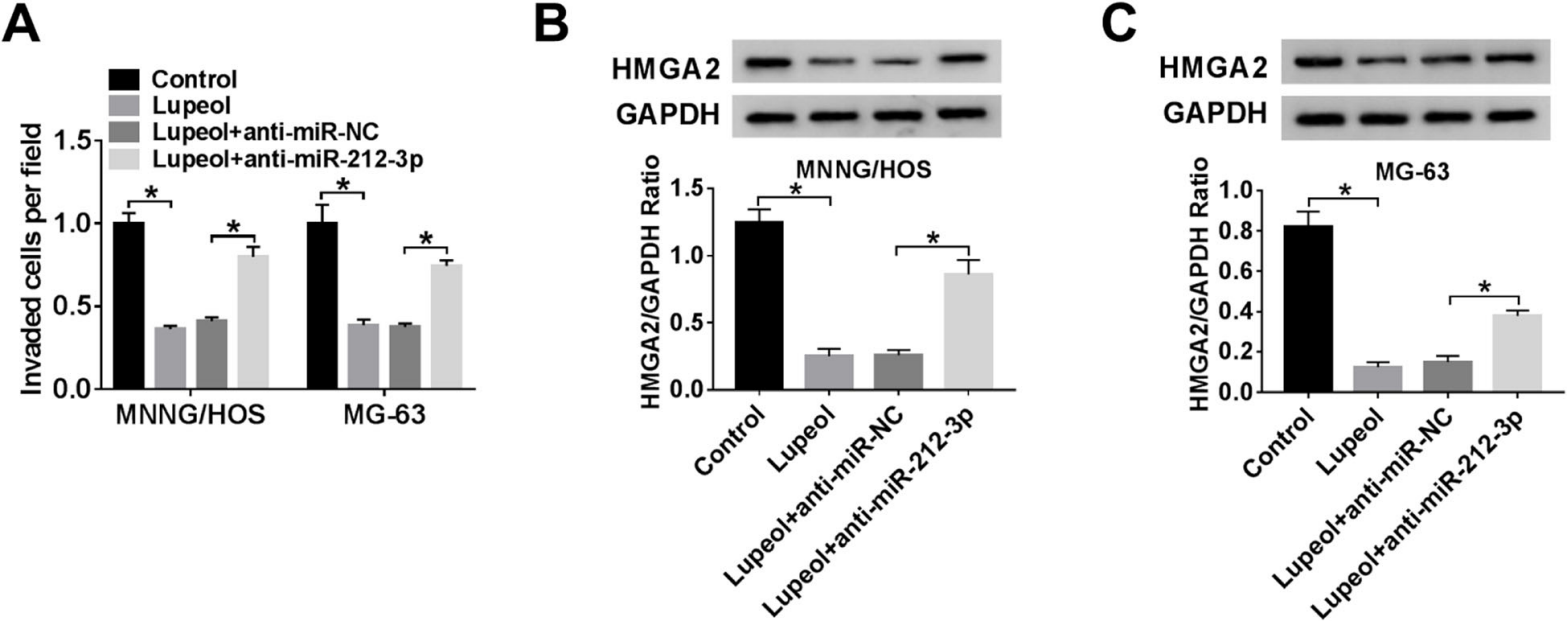
Lupeol mediated the expression of HMGA2 via regulating miR-212-3p. MNNG/HOS and MG-63 cells were transfected with anti-miR-NC or anti-miR-212-3p. **a** Transwell invasion was used to evaluate cellular invasive rate after lupeol treatment in MNNG/HOS and MG-63 cells. **b**, **c** The protein expression of HMGA2 in MNNG/HOS and MG-63 cells with or without lupeol treatment was detected by western blot. ^*^P < 0.05, *n* = 3

To verify whether the inhibitory action of lupeol on OS development was mediated by regulating the miR-212-3p/HMGA2 axis, transwell assay and western blot assay were used to evaluate invasion and the protein expression level of HMGA2 in MNNG/HOS and MG-63 cells, respectively. As shown in Fig. 6a, the silencing of miR-212-3p recovered invasion ability of the two cell lines, which was inhibited by lupeol. Meanwhile, a high expression level of HMGA2 (Fig. 6b, c) in Lupeol + anti-miR-212-3p were observed in vitro.

## Discussion

Osteosarcoma (OS) is one of the severe diseases that endanger the health of the world’s population. Despite many efforts to improve surgery, chemotherapy, and postoperative adjuvant chemotherapy, its rate of death remains high by now [22]. In addition, it is still unclear about understanding on biomarker and signaling pathway of cells derived from patients with OS in the initial phase [23], and traditional chemotherapy therapy contributes to resistance and side effects for patients with OS. Thereby, many researchers have been concentrating on exploring novel ingredients from natural products for therapy of OS. Lupeol is an anti-OS natural ingredient in plants and has been reported for the biological activities on anti-cancer and anti-inflammation in the past 25 years [24]. A previous paper reported that lupeol inhibited migration and invasion of OS cells by regulating p38/MAPK and PI3K/Akt signaling pathways in vitro [13]. However, the potential tumor-against effect of lupeol remains unclear. Thus, to find underlying targets for OS remedy and explore the anti-cancer mechanisms of lupeol are particularly essential.

Firstly, the effects of different lupeol concentrations fora range of determining times on cell viability in OS cell lines were examined. The mRNA expression level of miR-212-3p was downregulated as lupeol concentration increased, further confirming the relationship between lupeol and miR-212-3p. We also verified the positive anti-tumor effects of lupeol administration on OS cells, consistent with previous reports [13]. In addition, miR-212-3p mimics was transfected into MNNG/HOS and MG-63 cells, and we found that it shared uniform effects as lupeol on inhibiting tumor cells, which could be enhanced by the treatment of lupeol partly.

We speculated that lupeol might exert its OS-suppressed function by regulating the expression of miR-212-3p. To verify this hypothesis, we silenced miR-212-3p in OS cells treated with lupeol and found that cell viability and invasion were facilitated, and apoptosis was repressed by silencing of miR-212-3p. These data also confirmed that the silencing of miR-212-3p partly attenuated the inhibitory effects of lupeol on OS cells.

A previous paper reported that overexpression of HMGA2 in NSCLC could serve as a molecular marker in the progression of lung cancer [25]. D’Angelo et al. found thatHMGA2 activation played a critical role in pituitary tumorigenesis, and it was negatively related to a set of miRNA [26]. Our data revealed that miR-212-3p directly targeted HMGA2 and suppressed its expression. In OS cells co-transfected with HMGA2 and miR-212-3p mimic, cell viability and invasion were enhanced compared with cells transfected with miR-212-3p only in OS cells. In contrast, the ability of apoptosis was reduced compared with transfection with miR-212-3p only in OS cells. Thus, we proposed that miR-212-3p regulated viability, apoptosis, and invasion of OS cells by regulating HMGA2.

Finally, we detected the expression level of HMGA2 with lupeol administration by gain- and loss-of-function of miR-212-3p. It was also confirmed that lupeol exerted a tumor-suppressor role in OS through the miR-212-3p/HMGA2 axis.

There still exist several limited in this research. For instance, we just focused on the two different OS cell lines without any animal model experiment. In addition, this research did not refer to clinical experiments.

## Conclusion

In conclusion, we identified that lupeol was a repressor of the tumor by regulating the expression of miR-212-3p, which targeted HMGA2, and miR-212-3p functioned as an oncogene in OS progression. The uncovered miR-212-3p/HMGA2 axis may provide a thoughtful therapeutic method partly for the treatment of OS.

## Data Availability

The datasets used or analyzed during the current study are available from the corresponding author on reasonable request.

## Abbreviations

OS: Osteosarcoma
HMGA2: High-mobility group AT-hook 2
qRT-PCR: Quantitative real-time polymerase chain reaction
miRNAs: MicroRNAs
HMG: High-mobility group
SDS-PAGE: Sodium dodecyl sulfate polyacrylamide gel electrophoresis
HRP: Horseradishperoxidase
SD: Standard deviation

## References

[1] Thompson LD (2013). Osteosarcoma. Ear Nose Throat J..

[2] Bramer JA (2009). Prognostic factors in localized extremity osteosarcoma: a systematic review. Eur J Surg Oncol..

[3] Weeden S (2001). The effect of local recurrence on survival in resected osteosarcoma. Eur J Cancer..

[4] van Maldegem AM (2012). Comprehensive analysis of published phase I/II clinical trials between 1990-2010 in osteosarcoma and Ewing sarcoma confirms limited outcomes and need for translational investment. Clin Sarcoma Res..

[5] Saleem M (2005). Lupeol, a fruit and vegetable based triterpene, induces apoptotic death of human pancreatic adenocarcinoma cells via inhibition of Ras signaling pathway. Carcinogenesis..

[6] Imam S (2007). Two triterpenes lupanone and lupeol isolated and identified from Tamarindus indica linn. Pak J Pharm Sci..

[7] Geetha T, Varalakshmi P (2001). Anti-inflammatory activity of lupeol and lupeol linoleate in rats. J Ethnopharmacol..

[8] Saleem M (2009). Lupeol, a novel anti-inflammatory and anti-cancer dietary triterpene. Cancer Lett..

[9] Lambertini E (2005). Expression of estrogen receptor alpha gene in breast cancer cells treated with transcription factor decoy is modulated by Bangladeshi natural plant extracts. Oncol Res..

[10] Saleem M (2009). Lupeol inhibits proliferation of human prostate cancer cells by targeting beta-catenin signaling. Carcinogenesis..

[11] Lee TK, et al. Correction: lupeol suppresses cisplatin-induced nuclear factor-kappaB activation in head and neck squamous cell carcinoma and inhibits local invasion and nodal metastasis in an orthotopic nude mouse model. Cancer Res. 2016;76(7):2052–3. 10.1158/0008-5472.CAN-16-0381 27197241

[12] Tarapore RS (2010). Specific targeting of Wnt/beta-catenin signaling in human melanoma cells by a dietary triterpene lupeol. Carcinogenesis..

[13] Hsu MJ, et al. Lupeol suppresses migration and invasion via p38/MAPK and PI3K/Akt signaling pathways in human osteosarcoma U-2 OS cells. Biosci Biotechnol Biochem. 2019;83(9):1729-39. 10.1080/09168451.2019.1606693 31010399

[14] Croce CM, Calin GA (2005). miRNAs, cancer, and stem cell division. Cell..

[15] Luo XJ (2014). MicroRNA-212 inhibits osteosarcoma cells proliferation and invasion by down-regulation of Sox4. Cell Physiol Biochem..

[16] Lam K (2014). Hmga2 is a direct target gene of RUNX1 and regulates expansion of myeloid progenitors in mice. Blood..

[17] Komuro A (2018). Identification of a novel fusion gene HMGA2-EGFR in glioblastoma. Int J Cancer..

[18] Liu W (2015). MicroRNA-490-3p regulates cell proliferation and apoptosis by targeting HMGA2 in osteosarcoma. FEBS Lett.

[19] Liu Y (2016). Lupeol induces apoptosis and cell cycle arrest of human osteosarcoma cells through PI3K/AKT/mTOR pathway. Technol Cancer Res Treat..

[20] Liu Q (2017). Downregulation of long noncoding RNA TUG1 inhibits proliferation and induces apoptosis through the TUG1/miR-142/ZEB2 axis in bladder cancer cells. Onco Targets Ther..

[21] Liang H (2018). LncRNA PTAR promotes EMT and invasion-metastasis in serous ovarian cancer by competitively binding miR-101-3p to regulate ZEB1 expression. Mol Cancer..

[22] Cao J (2017). TUG1 promotes osteosarcoma tumorigenesis by upregulating EZH2 expression via miR-144-3p. Int J Oncol.

[23] Cai L (2017). The lnc RNA HNF 1A-AS 1 is a negative prognostic factor and promotes tumorigenesis in osteosarcoma. J Cell Mol Med.

[24] Gallo MB, Sarachine MJ (2009). Biological activities of lupeol. Int J Biomed Pharm Sci..

[25] Meyer B (2007). HMGA2 overexpression in non-small cell lung cancer. Mol Carcinog..

[26] D'Angelo D (2012). Altered microRNA expression profile in human pituitary GH adenomas: down-regulation of miRNA targeting HMGA1, HMGA2, and E2F1. J Clin Endocrinol Metab..

